# Chromosomes of three gall wasps of the tribe Aylacini (Hymenoptera, Cynipidae)

**DOI:** 10.3897/compcytogen.v15.i2.66781

**Published:** 2021-06-04

**Authors:** Vladimir E. Gokhman

**Affiliations:** 1 Botanical Garden, Moscow State University, Moscow 119234, Russia Moscow State University Moscow Russia

**Keywords:** *Aulacidea hieracii*, chromosome, Cynipidae, dicentric, gall wasps, *Isocolus jaceae*, *Isocolus scabiosae*, karyotype

## Abstract

Chromosomes of two species of the tribe Aylacini (Cynipidae), *Isocolus jaceae* (Schenck, 1863) and *I. scabiosae* (Giraud, 1859) (both have 2n = 18) were studied for the first time. In addition, 2n = 20 is confirmed in a member of the same tribe, *Aulacidea hieracii* (Bouché, 1834). All chromosomes of these gall wasps are biarmed; however, they gradually decrease in size in the case of *A. hieracii*, whereas a pair of large metacentrics is characteristic of karyotypes of both *Isocolus* Förster, 1869 species. Chromosomes of the two latter gall wasps are either metacentric or submetacentric, but elements with lower centromeric indices prevail in the karyotype of *A. hieracii*. Chromomycin A_3_ (CMA_3_)/DAPI staining revealed single CMA_3_-positive bands on a particular pair of chromosomes of all species, and these bands apparently refer to the nucleolus organizing regions (NORs). However, localization of CMA_3_-positive bands differs substantially between the studied members of *Isocolus* and *Aulacidea* Ashmead, 1897. Together with normal haploid and diploid mitotic divisions, several metaphase plates with 2n = 17 containing a peculiar dicentric chromosome were found in a single male specimen of *I. scabiosae*; this appears to be the first report of an obvious dicentric in the order Hymenoptera in general. Certain aspects of the chromosome diversity and karyotype evolution within the family Cynipidae and the tribe Aylacini in particular are briefly discussed.

## Introduction

Parasitoid Hymenoptera is an extremely species-rich, taxonomically complicated and economically important insect group (Forbes et al. 2018). The overwhelming majority of this group attack insects and some other arthropods; however, certain taxa of the ‘parasitoid’ Hymenoptera are in fact secondarily phytophagous (Quicke 1997). Among these herbivores, gall wasps of the family Cynipidae are the most diverse, with their world fauna exceeding 1400 species (Huber 2017). The tribe Aylacini s.l. (Ronquist et al. 2015) was previously considered a paraphyletic assemblage of the least advanced members of the monophyletic Cynipidae (Nieves-Aldrey 1994; Melika 2006). However, the recent analysis by Blaimer et al. (2020) shows that other cynipoid families render the latter group paraphyletic, recovering Aylacini as a basal monophyletic lineage of Cynipidae s.str.

Chromosomes of approximately 30 species of the family Cynipidae s.l. (sensu Blaimer et al. 2020) have been studied up to now (Dodds 1938; Sanderson 1988; Abe 1994, 1998, 2006; Gokhman et al. 2015). However, many aspects of karyotype evolution of gall wasps remain unknown due to lack of data on chromosome sets of many groups, especially basal ones. Specifically, karyotypes of only two members of the Aylacini, i.e., *Xestophanes potentillae* (Retzius, 1873) and *Aulacidea hieracii* (Bouché, 1834), are known at present (Dodds 1938). Moreover, chromosomes of these gall wasps were examined in the late 1930s, and therefore only chromosome numbers and other general features of karyotype structure were described. In addition, chromosomes of the large aylacine genus *Isocolus* Förster, 1869 which is most closely related to *Aulacidea* Ashmead, 1897 (Melika 2006), remained unknown up to now. We managed to study chromosomes of *A. hieracii* as well as of two *Isocolus* species, *I. jaceae* (Schenck, 1863) and *I. scabiosae* (Giraud, 1859) using chromosome morphometry and staining with base-specific fluorochromes. The results of this work are given below.

## Materials and methods

Achene galls of *I. jaceae* as well as stem galls of *I. scabiosae* and *A. hieracii* were recovered from *Centaurea scabiosa* Linnaeus, *C. stoebe* Linnaeus and *Hieracium robustum* Fries (Asteraceae), respectively, in the wild in European Russia. Specifically, these galls were collected in Moscow (55°28'N, 36°52'E), the Dubovsky District of the Volgograd Province (49°01'N, 44°43'E) and in Saratov (51°33'N, 46°04'E) in 2019–2020 by V.E. Gokhman and M.I. Nikelshparg. After keeping the galls for about a month at 5 °C, immature stages of wasps were extracted from the dissected galls. Chromosomal preparations were obtained from cerebral ganglia and developing gonads of prepupae and early pupae, respectively, generally following the protocol developed by Imai et al. (1988) with a few modifications. Specifically, these organs were first dissected in 0.5% hypotonic sodium citrate solution containing 0.005% colchicine, and then transferred to fresh hypotonic solution and incubated for 30 min at room temperature. After that, the material was transferred onto a pre-cleaned microscope slide using a Pasteur pipette and then gently flushed with Fixative I (glacial acetic acid: absolute ethanol: distilled water 3:3:4). The tissues were disrupted using dissecting needles in an additional drop of Fixative I. A drop of Fixative II (glacial acetic acid: absolute ethanol 1:1) was applied to the center of the area, and the more aqueous phase was blotted off the edges of the slide. The slides were then dried for approximately half an hour and stored at room temperature. For the routine chromosome staining, the preparations were stained with a freshly prepared 3% Giemsa solution in 0.05M Sorensen’s phosphate buffer (Na_2_HPO_4_ + KH_2_PO_4_, pH 6.8) for a few hours.

Fluorochrome staining with chromomycin A_3_ and 4’, 6-diamidino-2-phenylindole (CMA_3_/DAPI) was performed according to Schweizer (1976) with certain modifications. Specifically, the slide was flooded with CMA_3_ staining solution (0.5 mg/ml in McIlvaine buffer containing 5 mM MgCl_2_), covered with a coverslip, and incubated in the dark for about ten days. The coverslip was then removed, and the slide was briefly rinsed with distilled water and air-dried. The slide was then flooded with DAPI solution (2 μg/ml in McIlvaine buffer), covered with a coverslip, and stained in the dark for 30 min. The coverslip was then removed, and the slide was briefly rinsed with distilled water before being air-dried. The preparation was then mounted in a mixture of glycerin and McIlvaine buffer (1:1) containing 2.5 mM MgCl_2_, and sealed with rubber cement. The slide was stored in the dark prior to examination for a minimum of three days.

Mitotic divisions were studied and photographed using an optic microscope Zeiss Axioskop 40 FL fitted with a digital camera Axiocam 208 color (Carl Zeiss, Germany). To produce illustrations, the resulting images were handled with the image processing programs ZEN version 3.0 (blue edition) and GIMP version 2.10. Mitotic chromosomes were measured on ten haploid metaphase plates of *A. hieracii* and *I. scabiosae* as well as on four diploid metaphase plates of *I. jaceae* using KaryoType software version 2.0 and then classified according to the guidelines provided by Levan et al. (1964).

## Results

**Figure 1.**
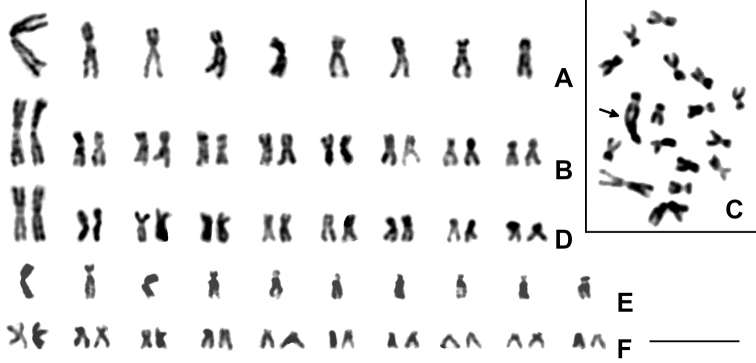
Mitotic chromosomes of Aylacini. *Isocolus scabiosae***A** haploid karyogram **B** diploid karyogram **C** diploid metaphase plate with dicentric chromosome (indicated by arrow); *I. jaceae***D** diploid karyogram; *Aulacidea hieracii***E** haploid karyogram **F** diploid karyogram. Scale bar: 10 μm.

**Figure 2.**
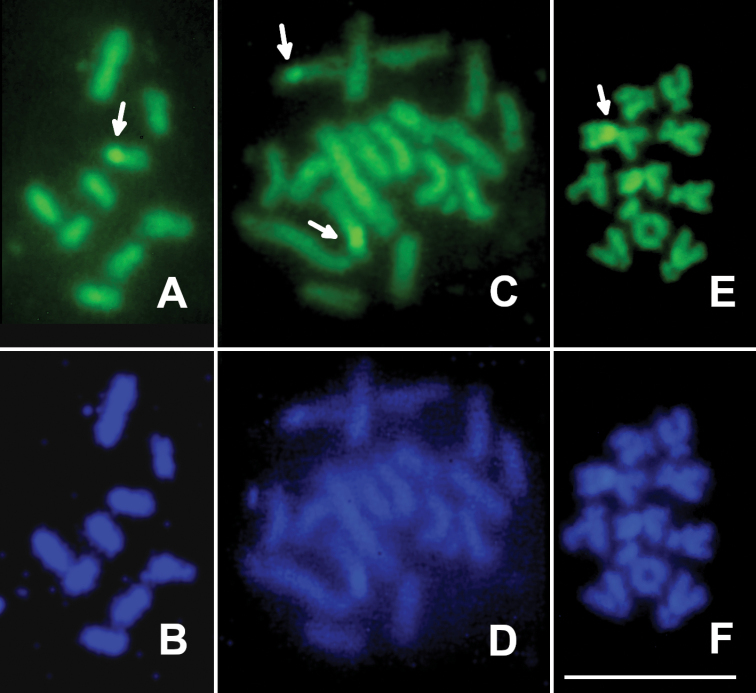
CMA_3_/DAPI staining of chromosomes of Aylacini. *Isocolus scabiosae* (haploid metaphase plate) **A**CMA_3_ staining **B**DAPI staining; *I. jaceae* (diploid metaphase plate) **C**CMA_3_ staining **D**DAPI staining; *Aulacidea hieracii* (haploid metaphase plate) **E**CMA_3_ staining **F**DAPI staining. Arrows indicate localization of CMA_3_-positive bands. Scale bar: 10 μm.

Males of *Isocolus scabiosae* generally have karyotypes with n = 9 (Fig. 1A), whereas females have 2n = 18 (Fig. 1B). The haploid chromosome set of this species harbors a large metacentric, with all other chromosomes (either metacentric or submetacentric) forming a more or less continuous gradation in length (Fig. 1A, B; Table 1). In addition, chromosome preparations obtained from testes of *I. scabiosae* contain a small proportion of apparently diploid cells. These cells can be found on analogous preparations from hymenopteran males of many species, but at least three metaphase plates from a particular male specimen of *I. scabiosae* had 2n = 17 and contained a characteristic dicentric chromosome (Fig. 1C). CMA_3_ reveals a single subterminal band on the shorter arm of a larger (apparently third) chromosome (Fig. 2A). This band obviously represents a nucleolus organizing region (NOR). On the other hand, DAPI produces uniform staining of all chromosomes (Fig. 2B).

**Table 1.** Relative lengths (RLs) and centromeric indices (CIs) of chromosomes of three species of the tribe Aylacini (mean ± SD).

Chromosome no.	*I. scabiosae*		*I. jaceae*		*A. hieracii*	
	RL	CI	RL	CI	RL	CI
1	19.54 ± 0.75	48.44 ± 1.35	18.87 ± 0.94	47.14 ± 1.67	12.52 ± 0.37	46.13 ± 2.00
2	11.52 ± 0.69	36.75 ± 2.10	11.73 ± 0.58	43.74 ± 2.50	11.95 ± 0.58	31.84 ± 3.41
3	11.36 ± 0.25	41.72 ± 3.87	11.16 ± 0.48	45.34 ± 3.61	11.02 ± 0.72	48.21 ± 1.79
4	10.60 ± 0.39	36.33 ± 3.33	10.80 ± 0.18	44.14 ± 3.44	10.61 ± 0.29	29.32 ± 2.06
5	10.27 ± 0.54	43.41 ± 3.60	10.25 ± 0.20	42.24 ± 5.18	10.01 ± 0.40	26.49 ± 2.92
6	9.69 ± 0.47	44.79 ± 3.48	9.93 ± 0.29	45.82 ± 2.83	9.37 ± 0.25	26.48 ± 3.88
7	9.48 ± 0.35	39.28 ± 3.07	9.77 ± 0.29	43.33 ± 4.29	9.16 ± 0.21	27.67 ± 2.71
8	8.96 ± 0.44	42.64 ± 3.72	9.29 ± 0.29	45.73 ± 1.69	8.73 ± 0.25	26.34 ± 3.46
9	8.58 ± 0.37	36.39 ± 3.80	8.20 ± 0.70	45.25 ± 5.42	8.45 ± 0.21	25.29 ± 3.79
10	–	–	–	–	8.18 ± 0.42	25.11 ± 3.66

Apart from *I. scabiosae*, only female specimens with 2n = 18 of *Isocolus jaceae* were found during the present study. The karyotype structure and fluorochrome staining of chromosomes of *I. jaceae* are similar to those of the previous species, probably except for chromosomes no. 2 and 4 which apparently have higher centromeric indices (Figs 1D, 2C, D; Table 1).

In *Aulacidea hieracii*, males and females have chromosome sets with n = 10 and 2n = 20, respectively (Fig. 1E, F). All chromosomes gradually decrease in size and are clearly biarmed (Table 1). Specifically, chromosomes no. 1 and 3 are metacentric, whereas chromosomes no. 2 and 4 are submetacentric. All other chromosomes are virtually intermediate between submetacentrics and subtelocentrics (Table 1). As in the first species studied in the present work, CMA_3_ visualizes a characteristic positive band on a single chromosome of the haploid set of *A. hieracii*. However, this band (an apparent NOR) is situated on the longer arm near the centromere of the first chromosome (Fig. 2E). As in *I. scabiosae* and *I. jaceae*, DAPI also does not reveal any bands on chromosomes of this species (Fig. 2F).

## Discussion

The above results show that *A. hieracii* and *I. scabiosae* are haplodiploid species, similar to bisexual generations of other Cynipidae studied in this respect (Sanderson 1988). As for *I. jaceae*, only female specimens were found during the present study, although males of this species are also known (Melika 2006).

The most frequent chromosome number in the family Cynipidae is n = 10 (Sanderson 1988), and this number is also confirmed for *A. hieracii*. Moreover, all chromosomes of the gall wasps studied in the present paper are biarmed. Despite karyotypes of many members of the Cynipidae s.str. sensu Blaimer et al. (2020) with n = 10 contain at least some biarmed chromosomes (Sanderson 1988), only acrocentrics were found in the chromosome sets of a few species, i.e. *Dryocosmus kuriphilus* Yasumatsu, 1951 (Abe 1994) and *Belonocnema kinseyi* Weld, 1921 (Gokhman et al. 2015, cited as *B. treatae* Mayr, 1881; see Zhang et al. 2021). Interestingly, both *Dryocosmus* Giraud, 1859 and *Belonocnema* Mayr, 1881 represent relatively basal lineages within their clades, i.e., within the *Neuroterus*-group and *Cynips*-group respectively (sensu Liljeblad et al. 2008). Based on these data, we previously suggested that this karyotype structure is likely to be ancestral at least for members of their common clade (Gokhman et al. 2015), i.e., the tribe Cynipini (Ronquist et al. 2015). On the other hand, both *Aulacidea* and *Isocolus* belong to a separate clade, namely Aulacideini, which, in turn, is part of Aylacini s.l. (Ronquist et al. 2015; Blaimer et al. 2020). Unfortunately, further karyotypic data for the latter group, except for the chromosome number of n = 10 for *Xestophanes potentillae* (Dodds, 1938), are absent. Nevertheless, we can assume at this point that a haploid karyotype containing ten biarmed chromosomes could be ancestral for Aylacini s.l. and perhaps even for the family Cynipidae s.str. in general. In this case, chromosome sets of other members of the latter clade which contain at least some acrocentrics, might represent derived character states.

A large metacentric found in both *Isocolus* species apparently originated via chromosomal fusion, and this feature can be a synapomorphy either of the whole genus or just of *I. jaceae* and *I. scabiosae* which are very close to each other in terms of morphology (Melika 2006). Since it is difficult both to separate *Isocolus* from *Aulacidea* and to distinguish species of the former genus, chromosomal characters may help improving taxonomy of these taxa. Independent chromosomal fusions similar to those found in *Isocolus* were previously detected in other cynipoids that belong to the family Figitidae (Gokhman et al. 2011).

A characteristic dicentric chromosome found in a particular male specimen of *I. scabiosae* apparently deserves special attention. To my best knowledge, this is the first report of an obvious dicentric with two visible primary constrictions, i.e., centromeres, in the order Hymenoptera in general. This chromosomal mutation was obviously deleterious (Barra and Fachinetti 2018), since it was restricted to a small fraction of spermatogonial divisions in a single individual which otherwise produced karyotypically normal sperm cells. In *I. scabiosae*, the dicentric chromosome apparently originated via telomeric fusion of two smaller submetacentrics.

The present study also revealed single putative NORs in the haploid karyotypes of *I. scabiosae* and *A. hieracii*, in addition to the only paired NOR in the diploid set of *I. jaceae*. Among other Cynipidae, similar results were obtained for *Diplolepis rosae* (Linnaeus, 1758) using FISH with 18S rDNA probe (Gokhman et al. 2014) as well as for *B. kinseyi* (Gokhman et al. 2015). Taken together with the same number of NORs in other studied Cynipoidea, including members of the family Figitidae (Gokhman et al. 2016), presence of the single NOR is therefore characteristic of the Cynipoidea in general. However, localization of this region can substantially vary between different members of the superfamily (see also Gokhman et al. 2016).
